# A Randomized, Comparative Study of Skin Adhesion Between CATHEREEPLUS Pad and Tegaderm Pad Film Dressings in Healthy Participants

**DOI:** 10.7759/cureus.72600

**Published:** 2024-10-29

**Authors:** Shiori Sakurai, Yuji Kawamura, Eri Nohmi, Takemasa Kokubo, Takashi Koikeda

**Affiliations:** 1 Medical Regulatory Affairs, NICHIBAN Co. Ltd., Tokyo, JPN; 2 Quality Assurance, NICHIBAN Co. Ltd., Tokyo, JPN; 3 Dermatology, Shiba Palace Clinic, Tokyo, JPN

**Keywords:** absorbent pad, adhesion to skin, dermal application, healthy participant, itchiness, skin reaction, transparent film adhesive dressing

## Abstract

Objective

This study aimed to compare the adhesion of CATHEREEPLUS_TM_ Pad (CPSP; NICHIBAN Co., Ltd., Tokyo, Japan) and Tegaderm^TM^ +Pad (TGMP; 3M, Maplewood, MN, USA) film dressings on the forearm skin of healthy participants over a four-day application period.

Methods

Twenty-six randomly assigned participants received CPSP dressing on one arm and TGMP on the other. The primary endpoint was adhesion to the skin after four days of dermal application. Secondary endpoints were adhesion and itchiness during the application period, pain experienced during dressing removal, skin maceration, adhesive residue immediately post-dressing removal, and skin reactions at one and 24 hours post-dressing removal. All endpoints were evaluated using a five- or six-point scoring system.

Results

Following four days of dressing application, 77% of participants in the CPSP group and 73% of those in the TGMP group scored 4 (most) or 5 (complete) for adhesion. No clinically significant problems such as itchiness, pain, skin maceration, adhesive residue, or skin reactions were observed in either group. No statistically significant differences in any of the endpoints were observed between the two groups.

Conclusion

Both CPSP and TGMP dressings showed good adhesion to the skin during four days of dermal application in healthy participants, with no significant difference in adhesion observed between the two groups.

## Introduction

Wound dressings are primarily used in wound care. They provide a barrier to prevent contamination, alleviate pain, and insulate wounds. Wound healing is an active and complex process that requires optimal conditions to facilitate healing [1]. Technological advancements have led to the development of >3,000 products designed to treat various wound types and to address different stages of the healing process [1]. Modern wound dressings have been developed and widely used in wound care. These dressings create a moist environment to promote healing and comprise hydrocolloids, alginates, and hydrogels [2]. Semipermeable film dressings consist of transparent, sticky polyurethane permeable to water vapor, oxygen, and carbon dioxide [3]. They also facilitate autolytic wound cleaning and prevent bacterial infections. Conventional dressings such as sterile gauze pads are used to pack open wounds, drawing fluid and exudate away from the wound, with their absorbent fibers acting as a filter [2]. Sterile gauze dressing changes are necessary to prevent the softening of healthy underlying tissues and are generally reported to be less cost-effective than modern wound dressings [4].

A semipermeable film wound dressing combined with a sterile gauze pad, such as a CATHEREEPLUS_TM_ Pad (CPSP; NICHIBAN Co., Ltd., Tokyo, Japan) or Tegaderm^TM^ +Pad (TGMP; 3M, Maplewood, MN, USA) film dressing, is used to cover and protect wounds, promote skin closure of wound edges, and support the injured area of the body. These dressings can be used during activities such as showering. In addition, these dressings are available for direct application at an injection or catheter insertion site to secure a dressing in place. Clinical evaluations of the TGMP have been undertaken involving patients requiring wound care in different situations, including obstetric and gynecological surgeries [5], median sternotomy wounds [6], and orthopedic surgeries [7,8]. However, no clinical evaluation of the CPSP dressing has been reported. Therefore, we aimed to compare the feasibility and safety of CPSP and TGMP in healthy individuals by assessing adhesion to the skin, itchiness during application, skin maceration, and skin reaction after removal of dressings in a four-day application of both CPSP and TGMP. The null hypothesis was that no differences would be observed in the assessments of the two dressings.

## Materials and methods

Study design

This randomized, comparative, parallel-group study evaluated adhesion after dermal application of CPSP and TGMP film dressings in healthy participants. In each group, the participants applied each dressing to intact skin on their left and right forearms for four days, with each participant applying the dressings simultaneously. This study was conducted at a single medical institute (SOUKEN Co., Ltd., Tokyo, Japan) in October 2023.

Ethics

The study was approved by the Institutional Review Board (Shiba Palace Clinic, Tokyo, Japan; Approval number: 152395-35242) on October 5, 2023, and was registered with the UMIN Clinical Trials Registry of Japan (UMIN study ID: UMIN000055805). All participants provided written informed consent prior to enrollment.

Study samples

The CPSP and TGMP study samples were purchased from a distributor. The overall sizes of the CPSP and TGMP film dressings were 80 × 120 mm and 90 × 100 mm, respectively. The pad sizes of CPSP and TGMP were 40 × 80 mm and 45 × 60 mm, respectively.

Study participants

The inclusion criteria comprised Japanese female and male participants aged 20-49 years, with no history of outpatient visits or medication for disease treatment and healthy skin, who were able to provide informed consent and shave forearm hair with a T-shaped razor, if necessary.

The exclusion criteria comprised participants with any of the following conditions: a history of previous treatment applied to the skin, particularly within the previous two weeks; a history of anaphylactoid or hypersensitivity symptoms when applying a skin dressing or bandage; a current history of atopic dermatitis; skin symptoms such as wounds or eczema on the forearm; or excessive hairiness on the forearm. All participants were screened by an investigator for skin assessment and eligibility verification.

Data collection

Participants visited the clinical institute on days one, five, and six. A medical expert applied the study samples to the participants on day one, removed them on day five, and examined the participants on day six, 24 hours post-dressing removal, in addition to examinations on days one and five. The participants recorded their self-assessment results from day one to day four evenings while living their everyday lives.

Endpoints

Primary Endpoint

The primary endpoint was adhesion to the skin four days after dressing application on day five, which was evaluated by the investigator using in-house score criteria.

Secondary Endpoints

Adhesion to the skin: Adhesion to the skin was evaluated at different time points after showering on days one to four for each participant.

Itchiness: Itchiness was evaluated at time points immediately after the application of dressings on day one, after showering on days one to four, and immediately prior to dressing removal.

Pain, skin maceration, and adhesive residue: Pain was evaluated during dressing removal on day five. Skin maceration and adhesive residues were evaluated immediately after dressing removal on day five.

Skin reaction: Skin reaction was evaluated immediately, at one hour, and at 24 hours after dressing removal by the investigator. The skin reaction score was based on the standardization of patch test results [9].

Sample size estimation

A sample size of 26 participants was calculated, giving a null proportion of 0.8, an alternative proportion of 0.6, a p-value of 0.05, a power of 0.9, a one-to-one randomization rate, and a dropout rate of 0.20.

Randomization and allocation

The participants were randomized into two groups at a ratio of 1:1. In group 1, CPSP was applied to the right forearm and TGMP was applied to the left forearm. In group 2, TGMP was applied to the right forearm and CPSP was applied to the left forearm.

Statistical analyses

The CPSP and TGMP groups comprised 26 participants (13 left and 13 right forearms in each group). Scoring data were compared between the two groups using a Fisher’s exact test and a 95% confidence interval for the proportion of participants scoring 4 (most) or 5 (complete) for adhesion on day five or a Wilcoxon signed-rank test for scores in relation to the secondary endpoints. All statistical analyses were conducted using SAS software (SAS Institute, Cary, NC, USA). Statistical significance was set at p < 0.05.

## Results

In total, 26 participants were enrolled and randomized into groups 1 and 2. None of the participants withdrew from the study, and all participants received a final assessment on day six. The study participants ranged in age from 23 to 49 years (mean ± standard deviation = 43.5 ± 6.8 years in females (n = 13) and 44.1 ± 5.9 years in males (n = 13), respectively). Twenty-three participants were aged 40-49 years, comprising 11 females and 12 males.

Primary endpoint

The adhesion assessment findings are shown in Table 1. The percentage of participants scoring 4 (most) or 5 (complete) for adhesion was 76.9% (20/26) in the CPSP group and 73.1% (19/26) in the TGMP group on day five. The difference in the proportion between the two groups was 3.9% (95% confidence interval = 24.7% to 32.0%). No statistically significant difference was noted in the proportion of participants who scored 4 or 5 for adhesion between the two groups (Fisher’s exact test, p = 1.000).

**Table 1:** Adhesion. CPSP: CATHEREEPLUS Pad; TGMP: Tegaderm +Pad; N: number of participants; -: no participants scored.

Score		Day 1			Day 2			Day 3			Day 4			Day 5		
		CPSP	TGMP	p-value	CPSP	TGMP	p-value	CPSP	TGMP	p-value	CPSP	TGMP	p-value	CPSP	TGMP	p-value
		N = 26	N = 26		N = 26	N = 26		N = 26	N = 26		N = 26	N = 26		N = 26	N = 26	
5	Complete	23 (86%)	21 (81%)	0.563	21 (81%)	18 (69%)	0.836	18 (69%)	15 (58%)	1.000	17 (65%)	11 (42%)	0.550	17 (65%)	13 (50%)	0.668
4	Most	3 (12%)	4 (15%)		2 (7.7%)	5 (19%)		5 (19%)	6 (23%)		4 (15%)	8 (31%)		3 (12%)	6 (23%)	
3	85% or over	-	1 (3.8%)		1 (3.8%)	2 (7.7%)		-	4 (15%)		-	4 (15%)		-	4 (15%)	
2	70% or over	-	-		-	1 (3.8%)		-	-		1 (3.8%)	1 (3.8%)		1 (3.8%)	1 (3.8%)	
1	50% or over	-	-		-	-		-	-		-	-		-	1 (3.8%)	
0	Under 50%	-	-		2 (7.7%)	-		3 (12%)	1 (3.8%)		4 (15%)	2 (7.7%)		5 (19%)	1 (3.8%)	

Secondary endpoints

Adhesion

The proportion of participants who scored 5 (complete) for adhesion decreased gradually from day one to day five in both groups (Table 1). From day one to day five, two to five participants in the CPSP group scored 0 (under 50%) for adhesion, and one to two participants in the TGMP group scored 0 for adhesion. Data points where the participants scored 0 for itchiness, pain, skin maceration, adhesive residue, or skin reactions were excluded from the analysis.

Itchiness

Itchiness assessment findings are presented in Table 2. During the application period, 11 participants in the CPSP group experienced slight itchiness, and three and one participants experienced mild and moderate itchiness, respectively. In the TGMP group, 13 participants experienced slight itchiness and two participants experienced mild itchiness. No significant difference was observed in the proportion of participants who experienced itchiness between the groups during the study period.

**Table 2:** Itchiness. CPSP: CATHEREEPLUS Pad; TGMP: Tegaderm +Pad; N: number of participants; -: no participants scored; NC: not calculated.

Score		Immediately post-application			Day 1			Day 2			Day 3			Day 4			Day 5		
		CPSP	TGMP	p-value	CPSP	TGMP	p-value	CPSP	TGMP	p-value	CPSP	TGMP	p-value	CPSP	TGMP	p-value	CPSP	TGMP	p-value
		N = 26	N = 26		N = 26	N = 26		N = 26	N = 26		N = 24	N = 26		N = 23	N = 25		N = 21	N = 25	
4	No findings	25 (96%)	25 (96%)	NC	22 (85%)	22 (85%)	1.000	19 (73%)	18 (69%)	1.000	17 (71%)	16 (62%)	0.727	17 (74%)	17 (68%)	1.000	18 (86%)	20 (80%)	1.000
3	Slight	1 (3.8%)	1 (3.8%)		4 (15%)	4 (15%)		6 (23%)	8 (31%)		6 (25%)	8 (31%)		4 (17%)	7 (28%)		2 (9.5%)	4 (16%)	
2	Mild	-	-		-	-		1 (3.8%)	-		1 (4.2%)	2 (7.7%)		1 (4.3%)	1 (4.0%)		1 (4.8%)	1 (4.0%)	
1	Moderate	-	-		-	-		-	-		-	-		1 (4.3%)	-		-	-	
0	Severe	-	-		-	-		-	-		-	-		-	-		-	-	

Pain, Skin Maceration, and Adhesive Residue

The pain, skin maceration, and adhesive residue assessment results are shown in Table 3. During dressing removal, one participant in the CPSP group experienced slight pain, while two participants in the TGMP group experienced slight pain. No skin macerations were observed in either group immediately post-dressing removal. A slight adhesive residue was noted on the skin of two participants in the TGMP group. No significant difference was observed in the proportion of participants experiencing pain, skin maceration, or adhesive residues on the skin between the groups.

**Table 3:** Pain, skin maceration, and adhesive residue. CPSP: CATHEREEPLUS Pad; TGMP: Tegaderm +Pad; N: number of participants; -: no participants scored; NC: not calculated.

Score		During removal			Immediately post-dressing removal			Immediately post-dressing removal		
		Pain			Skin maceration			Adhesive residue		
		CPSP	TGMP	p-value	CPSP	TGMP	p-value	CPSP	TGMP	p-value
		N = 21	N = 25		N = 21	N = 25		N = 21	N = 25	
4	No findings	20 (95%)	23 (92%)	NC	21 (100%)	25 (100%)	NC	21 (100%)	23 (92%)	0.500
3	Slight	1 (4.8%)	2 (8.0%)		-	-		-	2 (8.0%)	
2	Mild	-	-		-	-		-	-	
1	Moderate	-	-		-	-		-	-	
0	Severe	-	-		-	-		-	-	

Skin Reaction

The skin reaction assessment findings are shown in Table 4. At one hour after dressing removal, 10 and 11 participants in the CPSP and TGMP groups, respectively, reported slight erythema. At 24-hour post-dressing removal, only one participant in the CPSP group exhibited erythema, whereas the remaining participants showed no signs of erythema. In the TGMP group, at 24 hours post-dressing removal, only five participants exhibited slight erythema. No significant difference was observed in the number of participants who experienced skin reactions between the two groups at any time point.

**Table 4:** Skin reaction. CPSP: CATHEREEPLUS Pad; TGMP: Tegaderm +Pad; N: number of participants; -: no participants scored.

Score		Immediately post-dressing removal			1-hour post-dressing removal			24-hour post-dressing removal		
		CPSP	TGMP	p-value	CPSP	TGMP	p-value	CPSP	TGMP	p-value
		N = 21	N = 25		N = 21	N = 25		N = 21	N = 25	
5	No reaction	16 (76%)	22 (88%)	0.375	11 (52%)	14 (56%)	1.000	20 (95%)	20 (80%)	0.625
4	Slight erythema	5 (24%)	3 (12%)		10 (48%)	11 (44%)		-	5 (20%)	
3	Erythema	-	-		-	-		1 (4.8%)	-	
2	Erythema and papule	-	-		-	-		-	-	
1	Erythema, papule, and vesicle	-	-		-	-		-	-	
0	Coalescent vesicles	-	-		-	-		-	-	

## Discussion

This study aimed to compare the adhesion of CPSP and TGMP to the skin of 26 healthy participants during a four-day dermal application to the forearms. Additionally, itchiness during the four-day application period, pain experienced during dressing removal, skin maceration, adhesive residue immediately post-dressing removal, and skin reactions at one hour and 24 hours post-dressing removal were assessed.

The sex ratio of the study participants was equal; however, most participants were aged 40-49 years, with a smaller number of participants in their 20s and 30s. While skin elasticity and contractility, which are factors that may affect skin adhesion, decline with age, no significant differences have been reported in terms of skin elasticity and contractility between individuals aged in their 20s and 40s [10]. Therefore, although most participants in this study were aged 40-49 years, we consider that this did not influence the assessment outcomes in terms of the adhesion of the dressings to the skin.

To minimize the effect of incomplete data, we excluded all data points, except for adhesion, when the dressing was completely peeled off during the study period. This decision was made because data points, such as itchiness assessment, would be unreliable after dressing removal.

On day five, 21 and 25 participants in the CPSP and TGMP groups, respectively, had maintained their dressings and underwent all of the study assessments, providing sufficient sample sizes for data analysis. The number of participants with complete adhesion in the TGMP group increased from 11 on day four to 13 on day five (Table 1). This increase may have been because adhesion was evaluated by the study investigator on day five, whereas it had been evaluated by individual participants on day four. As the primary endpoint on day five was evaluated by the investigator, which we considered more reliable and objective, we determined that the study had no reliability issues. After four days of dermal application, 77% of the participants in the CPSP group and 73% in the TGMP group scored 4 (most) or 5 (complete) for adhesion. However, the differences were not statistically significant. Both CPSP and TGMP film dressings demonstrated good skin adhesion during four days of dermal application. CPSP and TGMP film dressings are widely used for patients requiring wound care. Concerning the TGMP, clinical evidence suggests this dressing type is useful in various situations involving obstetric and gynecological surgeries after cesarean section, hysterectomy, or laparotomy [5], median sternotomy wounds for coronary artery bypass grafting [6], or orthopedic surgeries involving the hip or knee [7,8]. According to clinical studies, TGMP heals wounds and prevents surgical site infections in the abdomen [5], chest [6], hip, and knee [7,8]. No clinical assessment in patients using a CPSP film dressing has been reported; however, we consider that the CPSP is clinically useful for wound management in the abdomen, chest, hip, or knee in patients, similar to the TGMP, because the CPSP had the same adhesion as the TGMP for four days consecutively in this study.

This study had some limitations. The study was conducted on the forearm, which may have different adhesion properties compared with regions with greater skin distensibility or over joints. Therefore, these findings may not be generalizable to all anatomical sites. The dressing sizes used in this study were limited, and results may differ using larger and smaller dressings. Further research is needed to investigate the effect of these variables on the adhesion properties.

The Centers for Disease Control and Prevention guidelines recommend that gauze dressings used for short-term central venous catheters should be replaced every two days to prevent infection [11]. While CPSP and TGMP film dressings can be used for four consecutive days based on their adhesion, it is essential to promptly replace the dressings to prevent infection if they become soiled or exudate leaks out of the dressing.

Itchiness during the study period was noted in approximately half of the participants in the CPSP and TGMP groups, with all these participants reporting slight or mild itchiness, except for one participant in the CPSP group who experienced moderate itchiness on day four. Skin reactions post-dressing removal were observed in both groups. Slight erythema was more frequently noted at one hour post-dressing removal than immediately or at 24 hours post-dressing removal. Skin reactions were considered reversible changes. The skin reaction scores were low, except for erythema. Participants with moderate itchiness did not include those with erythema. No significant difference was observed in the number of participants who experienced itchiness or skin reactions between the two groups during the application period. The itchiness and skin reactions were not severe, and we considered that both film dressings have no clinically significant concerns regarding skin irritation during clinical use.

Dressing changes are known to cause discomfort, often resulting in pain [12], but the frequency of slight pain during dressing removal was low (4.8% (1/21) and 8.0% (2/25) in the CPSP and TGMP groups, respectively). This suggests that the risk of pain when removing the CPSP and TGMP is low in clinical use. A reasonable moisture permeability prevents excessive perspiration and provides a moist environment suitable for wound healing [3]. Excessive skin maceration during dressing application can lead to itching and wound deterioration. In this study, no skin maceration was noted four days after dressing application, suggesting that the CPSP and TGMP film dressings are suitably breathable because they are made of a highly permeable polyurethane film material. Patients may find it unpleasant when the adhesive residue remains on the skin after dressing removal [13], owing to its appearance and hygienic concerns. In this study, minimal adhesive residue was noted, suggesting that discomfort associated with adhesive residue is unlikely to be a significant issue when using the CPSP or TGMP film dressings. Based on secondary endpoint results, no clinically significant issues, including pain during dressing removal, skin maceration, adhesive residue immediately post-dressing removal, or skin reaction post-dressing removal, were noted in the two groups. No statistically significant differences were observed in any secondary endpoints between the two groups.

## Conclusions

This study aimed to evaluate the adhesion properties of CPSP and TGMP film dressings to the skin during a four-day application period on the forearms of healthy participants. The results indicated that both film dressings exhibited good adhesion to the skin over the four-day application period, with no significant differences in adhesion observed between the two groups.
